# Intraventricular CNS aspergillosis in a patient with prior history of COVID-19: Case report and review of literature

**DOI:** 10.1016/j.amsu.2022.104122

**Published:** 2022-07-07

**Authors:** Samuel Berchi Kankam, Hiva Saffar, Milad Shafizadeh, Shirin Afhami, Alireza Khoshnevisan

**Affiliations:** a Department of Neurosurgery, Shariati Hospital, Tehran University of Medical Sciences, Tehran, Iran; b Department of Pathology, Shariati Hospital, Tehran University of Medical Sciences, Tehran, Iran; c Department of Infectious Diseases, Shariati Hospital, Tehran University of Medical Sciences, Tehran, Iran; d International Neurosurgery Group (ING), Universal Scientific Education and Research Network (USERN), Tehran, Iran

**Keywords:** CNS, Aspergillosis, COVID-19, Intraventricular space, SARS-CoV-2, Case report, IVA, Intraventricular Aspergillosis, COVID-19, Coronavirus disease 2019, SARS-CoV-2, Severe Acute Respiratory Syndrome Coronavirus 2, CNS, Central Nervous System, IPA, Invasive Pulmonary Aspergillosis, GCS, Glasgow Coma Scale

## Abstract

**Introduction and importance:**

Although some immunocompetent patients have developed invasive aspergillosis, the vast majority of cases are seen in immunocompromised patients. COVID-19 infection has been proposed to cause immune dysfunction or suppression, which predisposes patients to fungal co-infections such as mucormycosis and aspergillosis.

**Case presentation:**

A 58-year-old woman was admitted to the hospital with confusion, dysarthria, and loss of consciousness. The patient had a 1-month prior history of severe COVID-19 infection. A computerized tomography (CT) scan and a magnetic resonance imaging (MRI) revealed an intraventricular lesion with perilesional edema and a significant midline shift, which was initially thought to be an intraventricular tumor. Following a posterior parietal craniotomy, the lesion was resected via a transcortical approach from the posterior parietal region to the right lateral ventricle. Histopathological findings confirmed intraventricular aspergillosis (IVA). The patient was treated with intravenous amphotericin B for two months and discharged with oral variconazole for 4 months.

**Discussion:**

Covid-19 infections can result in- dissemination of fungal diseases such as aspergillosis. As a minor component of cerebral aspergillosis with a poor prognosis, intraventricular aspergillosis necessitates prompt treatment, which includes surgical resection and the administration of anti-fungal medications.

**Conclusion:**

Infection with COVID-19 causes immune dysfunction, which leads to fungal co-infection, including CNS aspergillosis. As a result, all COVID-19 patients who present with acute neurologic symptoms should have CNS aspergillosis considered in their differential diagnosis.

## Highlights

• Infection with COVID-19 can cause immune dysfunction, which may lead to fungal co-infection, including CNS aspergillosis.
• Workup of COVID-19 patients with acute neurologic symptoms should be broad and include fungal infectious.
• Patients with CNS aspergillosis should receive prompt treatment because these cases have poor prognosis and can be fatal.

## 1 Introduction

Invasive aspergillosis is common in immunocompromised patients with hematologic cancers (leukemia) and organ transplantation, and it has a very high mortality rate. Although uncommon in comparison to pulmonary aspergillosis, CNS aspergillosis is usually fatal if not treated promptly. They frequently occur within the brain parenchyma [4] or less frequently, in the ventricles [7,10].

Since the beginning of the ongoing global pandemic caused by severe acute respiratory syndrome coronavirus 2 (SARS-CoV-2), COVID-19 infection has been shown to cause immune dysfunction or suppression, predisposing patients to bacterial and fungal co infections [5,8]. Although, several cases of COVID-19 and invasive pulmonary aspergillosis (IPA) [5], and mucormycosis [8] have been reported, no case of COVID-19 and intraventricular aspergillosis (IVA) has been reported. We present a patient with a history of COVID-19 who was referred for IVA and pulmonary aspergillosis and discuss diagnosis and management of IVA. This is the first reported case, to the best of our knowledge.

## 2 Case presentation

**Fig. 1:**
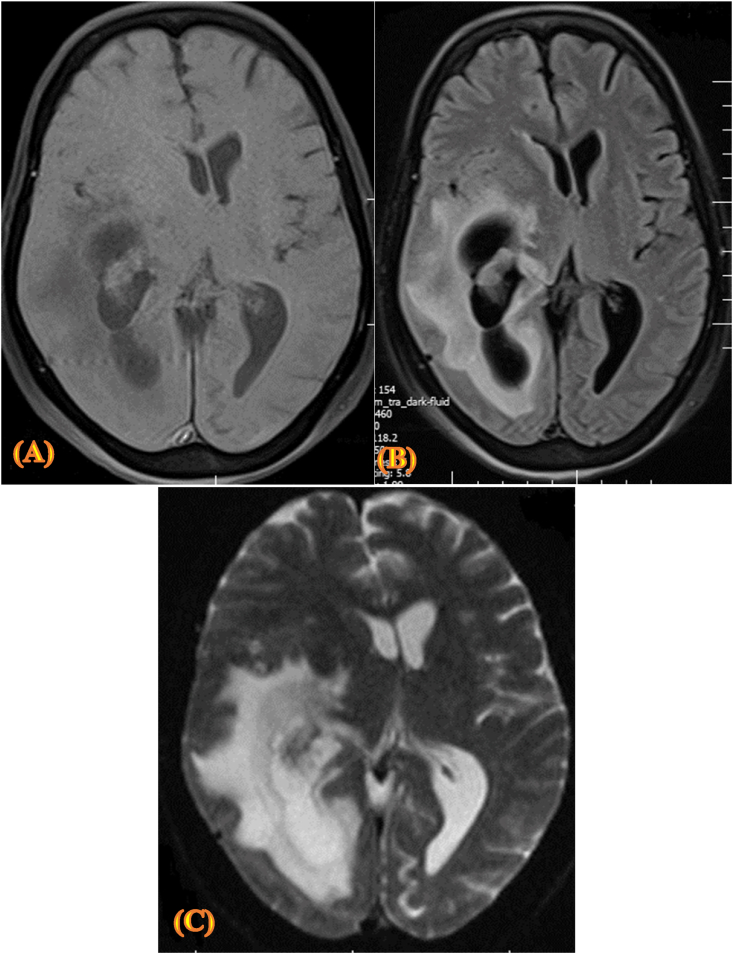
Preoperative MRI image, A; T1W, Axial view. Isointense lesion in right ventricle with midline shift. B; PD MRI image, Axial view. Perilesional edema is seen. C; T2W image. Axial view: Isointense lesion in the right ventricle with surrounding edema and midline shift. (MRI, magnetic resonance imaging; PD, proton density).

A 58-year-old woman presented to the emergency department our general hospital with a week of confusion, dysarthria, and stupor. The patient was hospitalized to the ICU ward and treated with corticosteroids among other medications for a serious COVID-19 infection she acquired a month earlier. Family and psychosocial history were negative. Physical examination revealed a patient who was ill, confused and disoriented, with a GCS of 9/15 (M5E2V2). A neurological examination left-sided hemiparesis and no papilledema. Motor examinations revealed that the upper and lower extremity muscle force of the left side were 2/5. Also, a positive plantar reflex was observed on the left foot. Her level of consciousness rapidly deteriorated a few hours after admission and neurosurgical consultation. The patient was immediately sent for a brain CT scan, which revealed a right hypodense intraventricular lesion with a midline shift. An MRI of the brain revealed a right intraventricular lesion that was isointense on T1&T2 weighted images. A large non-demarcated intraventricular lesion in the right lateral ventricle was observed, with perilesional edema, mass effect, and significant shift of the midline structures (Fig. 1).

**Fig. 2:**
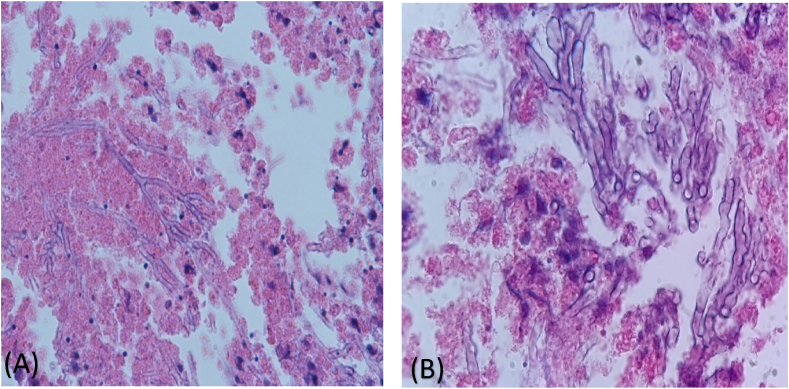
A: Foci of necrosis including numerous branching fungal hyphae with acute angle branching (H&E stain × 40). B: Septation with acute angle branching (H&E stain × 100).

The neurosurgeon (A.K) performed a posterior parietal craniotomy was on the patient. Following the opening of the dura, a transcortical approach from the posterior parietal region to the right lateral ventricle was made. The lateral ventricle's ependymal layer was opened, and a soft, greyish-colored lesion with slight hemorrhage and local invasion into the ependyma was discovered. Using microscopic surgical techniques with suction and bipolar diathermy, a macroscopic gross total resection was performed. Histopathological examination revealed necrotic foci with numerous branching fungal hyphae with acute angle branching. This finding was consistent with a fungal infection caused by Aspergillus species (Fig. 2).

## 3 Postoperative course

**Fig. 3:**
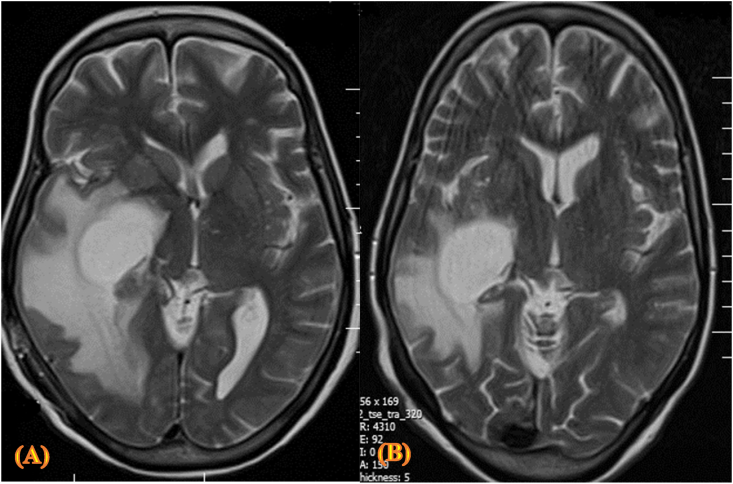
A. One-month postoperative MRI image is shown. Axial view of T2W: Edema persists, but the midline shift has decreased since the start of amphotericin B; B. Four-month postoperative MRI image. T2W Axial view: After 3 months of amphotericin B and voriconazole, edema and midline shift were reduced. (MRI, magnetic resonance imaging).

Following surgery, the patient's condition and level of consciousness improved. Based on the histopathological reports, the patient was immediately started on intravenous (IV) amphotericin B 250 mg daily, which she received for two months before being switched to oral voriconazole 400 mg Bid for additional 4 months. Her postoperative recovery has been uneventful, and she has completely recovered from her symptoms. (Fig. 3A and B -depict post-op MRI images changes between 1 and 4 months). This case report has been reported in line with the SCARE Criteria [14].

## 4 Discussion

Patients with severe COVID-19 infection, like other immunocompromised patients, are susceptible to invasive fungal infection, including mucormycosis and aspergillosis [5,8]. SARS-CoV-2, the primary cause of COVID-19, uses a variety of mechanisms to compromise and suppress the immune system. It reduces T-lymphocytes and increase the production of interleukins (IL-1 and IL-6), which destabilizes the immune system and creates an environment conducive for secondary infection with Aspergillus species [13]. Also, SARS-CoV-2, via a SARS spike (S1) protein, binds to angiotensin-converting enzymes 2 (ACE2) receptors expressed on epithelial cells of pneuomocytes and endothelial cells of blood vessels in brain, resulting in pulmonary infection and neurovascular damage, respectively [6,12]. Neurovascular inflammation provides an ideal environment for Aspergillus species to colonize. The invasion of the neurovascular system results in brain abscesses, thrombosis, meningitis, granuloma, vasculitis, cerebritis, and ventriculitis [4].

Notably, contrast-enhanced CT scans and MRI images are diagnostic but not specific for aspergillosis in cases of brain abscess [4]. Furthermore, hypodense and hyperdense images on CT scans and MRI images may be consistent with ischemic changes and cerebral infarction, respectively. Nonetheless, histopathologic examination including tissue and body fluids (blood and CSF) cultures continues to be the gold standard for diagnosis. Biopsied tissue samples stained with hematoxylin and eosin (H&E), Grocott-methenamine-silver (GMS) and periodic acid Schiff (PAS) may reveal the characteristic acute angle branching septate hyphae of Aspergillus species [3].

CNS aspergillosis is estimated to occur in 25–40% of disseminated aspergillosis cases [4]. Notably, CNS aspergillosis has also been reported in immunocompetent patients [3]. Although several cases of CNS aspergillosis have been reported in the literature [4,11], only six cases have been linked to intraventricular aspergillosis (IVA) [[1], [2], [3],^7^,^9^,^10^] (Table 1) and none to COVID-19 infection. This is the first reported case of a COVID-19 patient being referred for IVA, with subsequent investigation indicating IPA.

**Table 1:** Clinical manifestation, management and prognosis of intraventricular aspergillosis.

Cases/(year)	Age/Sex	Underlying condition	Symptom/Physical finding	Location of infection	Location of cerebral lesion	Hydrocephalus	Treatment			Outcome
							Surgery	Systemic fungal therapy	Duration of treatment	
Correa et al., /1975 [3]	49/F	None	Headache; vomiting; Kernig +/Brudzinski +; Bilateral papilledema	Brain (intraventricular mass)	Fourth ventricle	Present	Craniotomy, Resection	None	–	Died after 1 week
Morrow et al., /1983 [9]	36/M	Heroin abuser	Fever; Generalized Seizures; Neck stiffness	Postmortem finding consistent with ventriculitis	Not specified	Present	No surgery	No Antifungal therapy	–	Died after 40 days
Chen et al., /2010 [2]	39/M	Schizophrenia disease	Rapid decrease of LOC	Brain (intraventricular mass)	Right lateral ventricle	Present	Endoscopic ventriculostomy; Catheter drainage; VP shunt	Fluconazole; AmB, IV voriconazole (200 Bid)/2Week; Oral voriconazole (200 Bid)/2Week	1 month	Alive at 12 month F/U
Larijani et al., /2019 [7]	53/M	Renal transplantation	Rapid decrease of LOC, Bilateral papilledema	Brain (intraventricular mass)	Left lateral ventricle	Present	Endoscopic *trans*-middle frontal sulcus approach; Catheter drainage	Not specified	–	Died after 3 day during hemodialysis
Adachi et al., /2020 [1]	69/M	Acute lymphoblastic leukemia	Impaired LOC	Brain, multiple brain lesion; intraventricular brain abscess rupture	Right lateral ventricle	Absent	No surgery	L-AmB (5mg/kg/day); IV variconazole (12mg/kg/day)	Not specified	Died after 9 month with ALL relapse
Patel et al., /2020 [10]	59/M	Renal transplantation	Right-sided weakness; Speech difficulty	Lung (IPA); Brain (intraventricular mass)	Left lateral ventricle	Present	Catheter drainage; Surgical biopsy	Antifungal therapy not specified	Not specified	Not specified
Present cases/2022	58/F	COVID-19 infection; IPA	Fever; Confusion; Dysarthria; Rapid decrease of LOC; Babinski +	Lungs (IPA); Brain (intraventricular mass)	Right lateral ventricle	Absent	Craniotomy, Resection	IV AmB (3mg/kg/day) for 2 Months; Oral voriconazole 400 Bid (still on medication)	7 months	Alive at 7 months F/U

M, male; F, female; +, positive; -, not mentioned.

In 1975, Correa et al. described the first IVA, in which a lesion in the fourth ventricle blocked the foramina of Magendie, resulting in hydrocephalus [3]. Underlying conditions such as renal transplantation [7,10], heroin abuse [9], schizophrenia [2] and acute lymphoblastic leukemia (ALL) [1] has been reported. In this case, the underlying condition was COVID-19 infection (with corticosteroid medication). In agreement with one author [13], we believe COVID-19 infection resulted in an immunocompromised state, which led to secondary infections of IPA and IVA. In four of the cases [1,2,7,10], the isolated IVA lesion was found in the lateral ventricle. The rupture of an aspergillus brain abscess affected the intraventricular space indirectly in one case [1]. Despite the fact that hydrocephalus was observed in 5 of the cases [2,3,7,9,10], our patient did not have it. In all cases, histological examination and culture were used to make the diagnosis. Only one case, however, specified the Aspergillus species involved [9]. Among the six previous cases, 4 died with two case occurring within days after surgery and without any known explanation [3,7].

The prognosis of the CNS aspergillosis is generally abysmal. As a result, treatment should be aggressive and begin as soon as possible to improve patients' chances of survival. Surgical excision and intravenous amphotericin B administration are common treatments for CNS aspergillosis (including IVA). For improved patient outcomes, additional treatment options such as intraventricular administration of amphotericin B have been described [11]. Despite the fact that, like other studies [2,4], we attributed our patient's survival to early surgical excision and the administration of antifungal agents. We also believe that COVID-19 infection causes less immunosuppression or immune dysfunction than that seen in transplant patients or patients with hematologic malignancies who require immunosuppressive medications for a longer period of time to remain in remission. Furthermore, the ability to successfully treat patients with COVID-19, even in critical conditions, and the rapid evolution of vaccination are distinguishing features that distinguish patients with COVID-19 infection from other immunocompromised patients with CNS aspergillosis and/or IVA in terms of prognosis.

## 5 Conclusion

COVID-19 infection causes immune dysfunction, and it is possible to co-infect with bacterial and fungal agents, including Aspergillus species. As a result, in COVID-19 patients who exhibit confusion, loss of consciousness, seizures, or any neurological symptoms, aspergillosis should be considered in the differential diagnosis. Early administration of an antifungal agent such as amphotericin B may significantly improve patient outcome and prevent further neurologic deterioration and death.

## Provenance and peer review

Not commissioned, externally peer reviewed.

## Sources of funding

None.

## Ethical approval

Not required.

## Consent

Written Informed consent was obtained from the patient for publication of this case report and accompanying image. A copy of the written consent is available for review by the Editor-in-Chief of this journal on request.

## Author contributions

A.K, H.S: Conceived the idea.

S.B.K, M.S: Data collection, writing the paper.

A.K, S.B.K, S.A: Review and revised manuscript for intellectual content critically. All author approved the final version of the manuscript.

## Registration of research studies

1. Name of the registry:
2. Unique Identifying number or registration ID:
3. Hyperlink to your specific registration (must be publicly accessible and will be checked):

## Guarantor

Alireza Khoshnevisan.

## Declaration of competing interest

None.
